# Evidence for Reciprocal Structural Network Interactions Between Bilateral Crus Lobes and Broca’s Complex

**DOI:** 10.3389/fnana.2020.00027

**Published:** 2020-06-18

**Authors:** Hui Zhang, Yue Bao, Yuan Feng, Haijun Hu, Yibao Wang

**Affiliations:** ^1^Department of Neurosurgery, The First Affiliated Hospital of China Medical University, China Medical University, Shenyang, China; ^2^Department of Neurosurgery, Nanfang Hospital, Southern Medical University, Guangzhou, China; ^3^Department of Neurosurgery, Qingdao Municipal Hospital, School of Medicine, Qingdao University, Qingdao, China; ^4^Sleep Medicine Center, Nanfang Hospital, Southern Medical University, Guangzhou, China

**Keywords:** apraxia of speech, cerebro-cerebellar loop, motor–speech planning, mutism, tractography

## Abstract

While the proximal dentatothalamocortical tracts are considered pivotal in the occurrence of cerebellar mutism syndrome (CMS) after medulloblastoma resection, how the cerebellum participates in motor–speech networks through direct structural connectivity is still unclear. *Via* tractography, we provide evidence of cerebellar streamlines projecting into the left inferior frontal gyrus majorly connecting Broca’s complex and the bilateral Crus lobes. The streamlines, named Crus–Broca tracts, originated from the bilateral Crus lobes, synapsed onto the dentate nucleus, ascended into the superior cerebellar peduncle (where these streamlines were closely superior to the superior border of the supratonsillar cleft and the superolateral roof of the fourth ventricle), surprisingly bypassed the left red nucleus and the left thalamus, and ended at the subregions of Broca’s complex. The streamlines, named Broca–Crus tracts, originated from the subregions of Broca’s complex and ended predominantly at the right Crus lobes. If verified, the existence of these connections would support the notion of the bilateral cerebellums’ participation in motor–speech planning, and the anatomical relationship of Broca–Crus tracts with the supratonsillar cleft would merit consideration for further studies aimed at further elucidating CMS mechanisms.

## Introduction

A recent study including 370 pediatric medulloblastomas (MBs) reported that 23.8% of patients with an average age of 7.00 years (range 4.54–9.28 years) developed cerebellar mutism syndrome (CMS) after surgery (Jabarkheel et al., 2019). After alleviation of CMS, one third of the children have persistent dysarthria, while others exhibit residual phonological impairment (Tamburrini et al., 2015). Comparing with transvermian approaches (TrA), neurosurgeons preferentially use telovelar approaches (TeA) to resect MBs. However, both TrA and TeA are suitable for MBs in different locations (Tanriover et al., 2004; Ferguson et al., 2018) and surgical approach selection do not influence the risk of CMS (Renne et al., 2019). Previous analyses have indicated that midline MBs (Moxon-Emre et al., 2016), tumors extending to the rostral part of the fourth ventricle (Morris et al., 2009), and large tumors within the fourth ventricle (Rajesh et al., 2007) are highly associated with CMS. During exposure and resection of these tumors, the dentate nucleus (DN) and the superior cerebellar peduncle (SCP) are liable to be damaged regardless of the use of TrA or TeA (when the supratonsillar cleft was used in the case of the latter) (Rajesh et al., 2007; Akakin et al., 2014). Damage to the dentatothalamocortical tracts, which emerge from the hilus of the DN and immediately become part of the SCP, has been considered to be involved in the occurrence of CMS after resection of MBs (Morris et al., 2009; Korah et al., 2010). However, how the cerebellum participates in motor–speech networks through direct structural connectivity (namely, dentatocortical tracts) is still unclear (Tamburrini et al., 2015).

Although residual speech dysfunction after CMS is frequently defined as ataxic dysarthria, there is no consensus on the exact nature of the motor speech pathology underlying CMS (Korah et al., 2010; De Witte et al., 2017). In addition to deficits in motor speech execution, a variety of concomitant motor speech planning disturbances may manifest after alleviation of mutism, such as impaired verbal initiation, agrammatism, disrupted language dynamics, comprehension deficits, and reading and writing disorders (Di Rocco et al., 2011; De Smet et al., 2013). De Witte et al. (2017) reported only apraxia of speech (AoS) rather than ataxic dysarthria after pediatric MB surgery. AoS, which is defined as a dysfunction of both motor speech planning and programming, not only arises from injury to cerebral language-dominant motor speech regions (e.g., Broca’s area) (Dronkers, 1996; Hillis et al., 2004; Ottomeyer et al., 2009; Chang et al., 2015) but has also been observed in a number of cases of etiologically heterogeneous cerebellar disorders (Marien et al., 2006; De Smet et al., 2007; Marien and Verhoeven, 2007). Marien and Beaton (2014) suggested that both conditions possibly result from the disruption of a close functional interplay between the right cerebellum and the anterior motor speech region of the language-dominant hemisphere.

While it is well established that the cerebellum plays an important role in motor speech execution (Ackermann, 2008), the nature of any specific role for the cerebellum in motor speech planning—in terms of whether this role is direct and/or indirect—remains to be elucidated (Marien et al., 2015). A debate regarding whether or not the cerebellum sends fibers to Broca’s area dates back to at least the 1990s (Glickstein, 1993; Leiner et al., 1993). The roles of Broca’s area [which is comprised of Brodmann area 44 (BA 44) and BA 45] in speech production have become increasingly clear (Giahi Saravani et al., 2019; Klaus and Hartwigsen, 2019). Xiang et al. (2010) has proposed the concept of Broca’s complex (including BA 44, BA 45, and BA 47) with a possible division of labor for phonological, syntactic, and semantic planning. Unfortunately, recent studies have yielded inconsistent results regarding the existence of such a connection. In the Directions-Into-Velocities-of-Articulators neurocomputational model of speech acquisition and production, without an established connection between Broca’s complex and the cerebellum, the left ventral premotor cortex sends axonal fibers to the cerebellum, while the latter sends axonal fibers to the ventral motor cortex (Tourville and Guenther, 2011). Eickhoff et al. (2009) have suggested a hierarchically lower status of the cerebellum relative to the left premotor cortex and the anterior insular cortex in speech production based on their findings of an indirect cerebellar connection with BA 44 *via* the latter two areas.

Recently, advanced tractography studies have reconstructed portions/segments of these “inaccessible” long-range cerebro-cerebellar loops using advanced diffusion-based magnetic resonance imaging (dMRI) data from healthy subjects *via* DSI Studio (Meola et al., 2016), FSL’s diffusion toolbox (Mollink et al., 2016), and MRtrix3 (Pieterman et al., 2017). These techniques may be best suited to leveraging such multi-shell data in order to reconstruct the crossing fibers of the cerebro-cerebellar loops. However, unlike such high-resolution tractography in healthy subjects, the anatomical details of cerebro-cerebellar loops revealed in previous studies using clinical data from pediatric patients with posterior fossa syndrome have been comparatively coarse (Morris et al., 2009; Soelva et al., 2013). Therefore, in the present study, for the first time we focused on investigating the structural connections between the left inferior frontal gyrus (IFG), which includes Broca’s complex, and the cerebellum using public research data from healthy children (rather than using subjects with CMS) with the above-mentioned tractography software in an attempt to better elucidate the neural circuits that may subserve cerebellar roles in motor speech planning. Collectively, our findings may provide future directions for clinical research on the prevention of CMS.

## Materials and Methods

### Subjects and MRI Acquisition

Publicly available structural MRI (sMRI) and dMRI imaging data from all subjects of the HCP Lifespan Pilot project-1a^1^ were used in the present study. The modified acquisition protocol of these data has been demonstrated to be routinely feasible for the majority of healthy children (e.g., 8–9 years olds) to yield high-quality data^2^. The demographic details of these subjects are as follows: *n* = 26; ages included six 8–9 years old, six 14–15 years old, four 25–35 years old, five 45–55 years old, and five 65–75 years olds; in terms of sex, there were 14 females and 12 males; and handedness scores (in which the scores range from 0 to 8, where 0 = 100% left-handed and 8 = 100% right-handed) consisted of 0, 1, 3, 5, and 7 for one different individual for each of these scores, whereas the remaining 21 subjects each had a score of 8. The spatial resolution of the dMRI data was 1.5-mm isotropic. The *b* values were 1,500 and 2,500 s/mm^2^. The total number of diffusion sampling directions was 270. No experimental activity involving human subjects took place at the home institution of the authors. We received access to restricted data of this Human Connectome Project (HCP).

### Preprocessing of Multimodal Imaging Data

The preprocessing of dMRI and sMRI data was performed using the HCP pipelines (version 3.26.0) (Glasser et al., 2013). The preprocessing of dMRI mainly consisted of b0 image intensity normalization and susceptibility distortion, as well as eddy current and head motion corrections. The detailed steps of our preprocessing were as follows: (1) computed total readout time according to echo spacing, (2) normalized b0 image intensity across multiple series, (3) estimated the echo-planar imaging distortion using the ‘topup’ tool in FSL (Andersson et al., 2003) with the phase-encoding direction-reversed b0 images; (4) corrected eddy current-induced and head motion-induced distortions using the ‘eddy’ tool in FSL; (5) combined dMRI data with positive and negative phase-encoding directions using the ‘eddy_combine’ tool in FSL (Andersson and Sotiropoulos, 2015, 2016); and (6) averaged eddy-rotated ‘bvecs’ in FSL. The preprocessing of sMRI data mainly consisted of correcting magnetic resonance gradient non-linearity-induced distortions, cross-modal registration between T1 and T2, ‘acpc’ alignment with the original size and shape of the brain, and creation of each subject’s undistorted native-volume space. Finally, T1-weighted images were linearly registered to diffusion space using FSL’s ‘flirt’ tool with 12 degrees of freedom (Jenkinson et al., 2002).

### Creation of Volumetric Regions of Interest for Fiber Tracking

**FIGURE 1:**
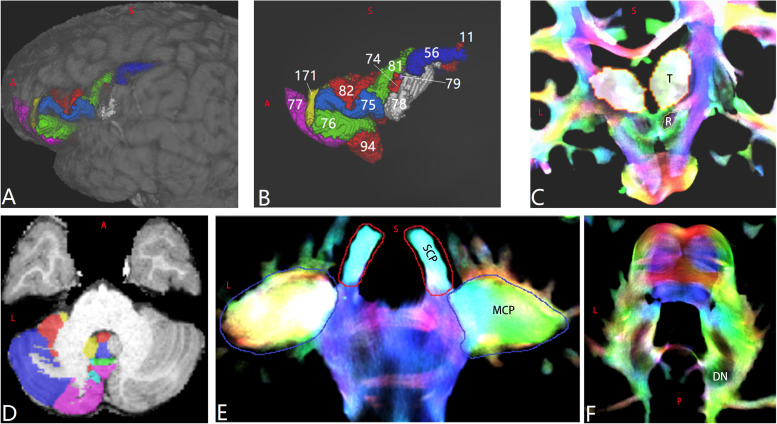
Regions of interest (ROIs) of a representative subject aged 8–9 years old (LS 2043). **(A,B)** Cerebral ROIs are defined by the Human Connectome Project (HCP) MMP 1.0 Atlas (HCP 66 and HCP 80 are not shown). **(C)** Bilateral thalamus (“T”) and red nucleus (“R”). **(D)** Part of the cerebellar ROIs defined by the spatially unbiased atlas for the cerebellum and brainstem atlas. **(E)** Bilateral superior cerebellar peduncle and middle cerebellar peduncle ROIs. **(F)** Bilateral dentate nucleus ROIs.

The left IFG was delimited by the inferior frontal sulcus, the lateral fissure, and the inferior precentral sulcus. Parcels corresponding to the defined left IFG in the HCP MMP 1.0 Atlas included the following in terms of parcel indices/area names: (1) Broca’s complex that included HCP 74-Area 44, HCP 75-Area 45, HCP 76-47l, HCP 77-a47r, HCP 171-p47r, HCP 66-47m, and HCP 94-47s; (2) ventral BA 6: HCP 11-PEF, HCP 56-6v, and HCP 78-6r; and (3) the inferior frontal sulcus: HCP 79-Area IFJa, HCP 80-Area IFJp, HCP 81-Area IFSp, and HCP 82-Area IFSa (Glasser et al., 2016). The regions of interest (ROIs) mentioned above in the diffusion space of each subject were attained with the following steps: In Freesurfer, ‘recon-all’ was performed on each registered T1 image (Dale et al., 1999); the annotation files of the HCP MMP 1.0 atlas were mapped from the fsaverage file to each subject’s space and were then transformed into the volumetric image (Figures 1A,B). In addition, the thalamus was segmented in Freesurfer with “segmentThalamicNuclei.sh” (Lglesias et al., 2018; Figure 1C). The volumetric ROIs of bilateral cerebellar lobules were attained based on a spatially unbiased atlas for the cerebellum and the brainstem (i.e., SUIT). The cerebellar cortex was subdivided into 28 ROIs, including 10 ROIs on each side of the cerebellar hemisphere and eight ROIs of the cerebellar vermis (Diedrichsen et al., 2009). The white matter regions contained in these 28 ROIs were excluded using a fractional anisotropy (FA)-based mask (FA ≤ 0.3) (Oh et al., 2009; Figure 1D).

Whole-brain tractography was performed using MRtrix3, as described previously (Palesi et al., 2017), until the desired total of 20 million streamlines had been selected. Track-density imaging (TDI) was attained using a 0.25-mm resolution grid (Calamante et al., 2010). The SCP and the middle cerebellar peduncle (MCP) (Figure 1E) were manually drawn on the coronal high-resolution TDI. The red nucleus (RN) on the coronal high-resolution TDI was recognized as a very hypointense region (Figure 1C; Calamante et al., 2013). The DN was drawn on the high-resolution TDI (Figure 1F). An ROI of the corpus callosum was manually drawn on the mid-sagittal slice of the FA image. Using “mrview” of MRtrix3 or Mricron, each ROI of each subject was carefully reviewed to ensure the accuracy of the anatomic registration and/or parcelation. For visualizing the main skeleton of these tracts at the group level, all the above-mentioned ROIs of subject LS 2043, one representative subject, were non-linearly registered to FSL_HCP1065_FA_1mm.nii.gz using the FNIRT tool of FSL 6.0.1.

### Tractography

Since the specific parameters of tractography can critically impact the results of tractography, we have included important parameters here to facilitate interested researchers in repeating the experiments and the analyses in our present study. Although tractography does not differentiate the direction of fiber bundles, a finely designed ROI approach based on the fiber pathway obtained from anatomical knowledge is usually used to limit the results of tractography. Therefore, through the pathway of reconstructed streamlines, their directions are determined. One method to delineate cerebello-cerebral tracts or cerebro-cerebellar tracts from other white matter fibers is to use the SCP (Palesi et al., 2015) or MCP (Palesi et al., 2017) as a ‘waypoint region,’ respectively. Considering that the ROIs of the HCP atlas are more related to specific functionalities and have smaller volumes (Glasser et al., 2016) compared to those of the SUIT atlas (Diedrichsen et al., 2009), we chose the ROIs of the left IFG as ‘seed regions’ in the tractography reconstruction of either cerebello-cerebral tracts or cerebro-cerebellar tracts. Therefore, the following ROI approach for the cerebellum–left IFG tracts (i.e., originating from the cerebellum and projecting in the left IFG) was used: (1) each ROI of the left IFG served as a ‘seed region,’ (2) each ROI among the 28 cerebellar ROIs served as an ‘end region’ in DSI studio, ‘waypoint mask’ in FSL, and ‘inclusion ROI’ in MRtrix3, (3) for the cerebellar-hemisphere ROI, the SCP ipsilateral to the cerebellar ROI was used as the ‘waypoint region’; for the cerebellar-vermis ROI, each side of the SCP was used as the ‘waypoint region’ (i.e., performed twice); and (4) the bilateral MCPs and corpus callosum ROI were used as the ‘exclusion regions’. For the left IFG-cerebellum tracts (i.e., originating from the left IFG and projecting in the cerebellum), the following ROI approach was used: (1) each ROI of the left IFG served as the ‘seed region’; (2) each ROI among the 28 cerebellar ROIs served as the ‘end region’ in DSI studio, ‘waypoint mask’ in FSL, and ‘inclusion ROI’ in MRtrix3; (3) for the cerebellar-hemisphere ROI, the MCP ipsilateral to the cerebellar ROI was used as the ‘waypoint region’; for the cerebellar-vermis ROI, each side of the MCP was used as the ‘waypoint region’ (i.e., performed twice); and (4) the bilateral SCPs and corpus callosum ROI were used as the ‘exclusion regions.’ In the preliminary experiments that reconstructed streamlines originating from the cerebellum and projecting in the left IFG using MRtrix3 software, we found that the vast majority of these cerebello-cerebral streamlines originated from four bilateral Crus lobes (SUIT 8-left Crus I, SUIT 11-left Crus II, SUIT 10-right Crus I, and SUIT 13-right Crus II, which were named as Crus ROIs thereinafter) and projected in five ROIs of Broca’s complex (HCP 74-BA 44, HCP 75-BA 45, HCP 76-47l, HCP 77-a47r, and HCP 171-p47r, which were named as Broca ROIs thereinafter) (Figure 1B). We had noted that the streamlines originating from Broca’s area and projecting in the cerebellum had been reconstructed by tractography in both infants (Pieterman et al., 2017) and adults (Palesi et al., 2017). Therefore, *via* the three tractography programs separately, we finally explored the characteristics of reciprocal structural network interactions between four Crus ROIs and five Broca ROIs, including the following: (1) the streamlines originating from each of four Crus ROIs and projecting in each of five Broca ROIs (i.e., 4 ^∗^ 5 tracts through the SCP, which we named as 20 Crus–Broca tracts) and (2) streamlines originating from each of five Broca ROIs and projecting in each of four Crus ROIs (i.e., 4 ^∗^ 5 tracts through the MCP, which we named as 20 Broca–Crus tracts). Here one tract denoted a structural connection between each of the four Crus ROIs and each of the five Broca ROIs. Its structural connectivity strength (SCS) was defined by the number of streamlines of the tract reconstructed via DSI Studio/Mrtrix3 or the number of nonzero non-zero voxels in the registered and binarized connectivity map of the tract *via* FSL (see below).

### Deterministic Tractography: DSI Studio

Deterministic tractography was performed in DSI studio^3^. The dMRI data were reconstructed using generalized q-sampling imaging, which can be applied to multi-shell data (Yeh et al., 2010). To explore the existence of a specific tract, the tractography process was performed using the following parameters: seed count = 500,000, topology-informed pruning = 0 (disabled), termination index = qa (Yeh et al., 2013), threshold = random, angular threshold = random, step size = random, smoothing = random, minimum length = 30 mm, maximum length = 300 mm, seed orientation = primary, seed position = subvoxel, randomize seeding = off, check ending = on, direction interpolation = trilinear, tracking algorithm = streamline (Euler) (Basser et al., 2000), default otsu = 0.6, differential tracking index = none, and differential tracking threshold = 0.2.

### Probabilistic Tractography: Diffusion Toolbox ‘Fdt’ of FSL 6.0.1

The diffusion directions at each voxel were modeled using ‘bedpostx_gpu’ (Behrens et al., 2007; Hernandez et al., 2013) as follows: fibers per voxel = 3, ARD weight = 1, burn in period = 1,000, number of jumps = 1,250, sample every = 25. The deconvolution model was used with sticks with a range of diffusivities. Because the data contained more than one non-zero *b* value, the deconvolution model we used was the ‘multi-shell model,’ in which the diffusion coefficient was modeled using a gamma distribution (Jbabdi et al., 2012). Fiber tracking was performed using “probtrackx2_gpu” (Hernandez-Fernandez et al., 2016) (with “-l -c 0.2 -S 2000 --steplength=0.5 -P 5000 --fibthresh=0.01 --distthresh=0.0 --sampvox = 0.0 --opd --wayorder” in the command line). The connectivity maps were binarized by 3.8 × 10^–5^ of the total number of samples. The latter equals the number of voxels of each seed ROI multiplied by 5,000 (Rilling et al., 2008).

### Probabilistic Tractography: MRtrix3

First, a tissue-segmented image appropriate for anatomically constrained tractography (Smith et al., 2012) was attained. Second, multi-shell and multi-tissue response functions were estimated. Third, multi-shell and multi-tissue constrained spherical deconvolutions, and estimations of fiber orientation distributions were performed using a harmonic order (kmax) of 8 (Tournier et al., 2007, 2008; Jeurissen et al., 2014). Fourth, tractography was performed using “tckgen” in the command line with the following options: -seeds 10,000,000 (given the aim of exploration of existence of a tract), -act, -backtrack, -maxlength 250, and other default parameters including -iFOD2 (Tournier et al., 2010).

### Postprocessing of Outputs of Tractography

**FIGURE 2:**
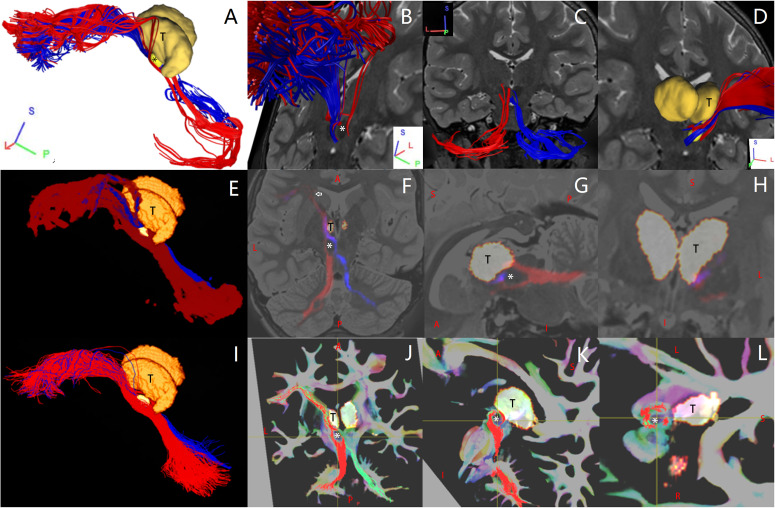
In one representative subject (LS 2043), the left (red) and the right (blue or green) Crus–Broca tracts reconstructed by DSI Studio **(A–D)**, FSL **(E–H)**, and MRtrix3 **(I–L)**. These tracts synapse on the ipsilateral dentate nucleus (DN), exit the hilus of the DN, ascend into the ipsilateral superior cerebellar peduncle, converge into one fasciculi around the left red nucleus (RN) and bypass the left RN and the left thalamus. The small bright yellow ellipsoid in **(A,D,E,I)** and the asterisk in **(A,B,F,G,J–L)** represents the left RN. The letter “T” denotes the left thalamus. The white arrow in **(F)** indicates the front end of the anterior isthmus of the left internal capsule.

If the streamlines from DSI Studio and MRtrix3 or non-zero voxels of the binarized connectivity map from FSL of each tract looped back on themselves or went through the cortical or the subcortical area of other cerebral regions on the halfway, for example, the superior frontal gyrus, or if the streamlines from DSI Studio and MRtrix3 did not end in Crus ROIs, they were deleted directly in DSI Studio, deleted using “tckedit” in MRtrix3, or were eliminated *via* re-performing “probtrackx2_gpu” in FSL with an additional personalized exclusion region. In fact, this step was not used often. Then, the SCS value of one tract was determined by the following: (1) the number of streamlines of the tract from DSI Studio/Mrtrix3, if it was not zero, and (2) the number of non-zero voxels in the binarized and registered connectivity map of the tract, if these voxels delineated at least the pathway of the tract between the DN and the front end of the anterior isthmus of the internal capsule (white arrow in Figure 2F). If not, the SCS value of the tract was set to zero, which did not imply that the tract did not exist at all in the subject, rather it simply denoted that the tract was not reconstructed. The reasons for the above-mentioned FSL criteria were as follows: As the probability connectivity distribution is propagated along a long pathway, its certainty in the connection probability decreases (Morris et al., 2008), so the FSL probabilistic approach is not as effective in reconstructing long pathways as streamline tractography (Khalsa et al., 2014). In addition, the binarization threshold for the probability connectivity distributions that we used in the present study is the strictest among all the ones reported previously (Rilling et al., 2008). In this case, the values of voxels belonging to both ends of these tracts in the connectivity maps would falsely not survive from the binarization threshold. For group-level visual inspections, the following steps were performed for Crus–Broca tracts and Broca–Crus tracts from all subjects: (1) transformed into voxel maps, (2) registered to a common space, and (3) added together within the same category of tract. These steps are important for understanding differences in image scales in related figures. In terms of Crus–Broca tracts, specifically the steps were as follows: (1) 20 Crus–Broca tracts of each subject from DSI Studio were added together using “Merge All” and were saved as an ROI (voxel) file; each Crus–Broca tract from MRtrix3 was transformed into a voxel map using “tckmap” (-map_zero, -precise); (2) outputs of step 1 and the binarized connectivity map of each Crus–Broca tract from FSL were registered to FSL_HCP1065_FA_1 mm.nii.gz using the FNIRT tool of FSL 6.0.1 (Jenkinson et al., 2002); and (3) outputs of step 2 from all subjects from each method were added together. Finally, we used the “tckmap” tool of MRtrix3 to map the streamline endpoints (including origin points and target points) to FA images at the subject and group level to explore the distribution patterns of these endpoints.

### Statistical Analysis

Statistical analyses were performed using SPSS 23.0 (SPSS, Chicago, IL, United States). Non-parametric analysis procedures were used to analyze 40 tracts from each subject. Independent–samples Mann–Whitney *U* tests were used to test for differences in SCS values of each tract between the 14 female and the 12 male subjects. The associations between the SCS values of all 40 tracts reconstructed separately by different methods were determined by Spearman rank–order correlations, and a bootstrapping method was used to attain 95% confidence intervals (CIs) of the median of each correlation coefficient (CC) with the following parameters: simple, 95% CI, 1,000 samples and percentile. Broca–Crus tracts and Crus–Broca tracts from all subjects were grouped by Broca ROIs (or Crus ROIs) separately. Then, related-samples Friedman’s two-way analysis of variance by ranks test followed by multiple pairwise comparisons were used to rank the contributions of each Broca ROI (or each Crus ROI) in Broca–Crus tracts or Crus–Broca tracts by comparing the mean ranks of the SCS values of these tracts grouped by Broca ROIs (i.e., treat = “Broca ROI,” block = “Crus ROI”; or for Crus ROIs: treat = “Crus ROI,” block = “Broca ROI”). The Kendall’s coefficient of concordance (k sample) was used to examine the degree of agreement among the three software programs in terms of their ranking contributions of each Broca ROI (or each Crus ROI) in Broca–Crus tracts or Crus–Broca tracts. Related-samples Wilcoxon signed-rank tests (two samples) were used to test for differences between the SCS values of Broca–left-Crus tracts and Broca–right Crus tracts, as well as between the SCS values of left- Crus–Broca tracts and right- Crus–Broca tracts (pairwise: left Crus I vs. right Crus I, and left Crus II vs. right Crus II). We assessed the asymmetries of Crus–Broca tracts and Broca–Crus tracts for all subjects and also for only subjects who were 8–9 years old. The Hodges–Lehmann 95% CI was used to determine the median of the differences in the asymmetry of Crus–Broca tracts or Broca–Crus tracts. Statistical significance was set at *p* < 0.05 for all analyses. Controlling the false discovery rate (FDR) of multiple comparisons was performed by the Benjamini–Hochberg procedure, which limited alpha errors to 5% (Benjamini and Hochberg, 1995).

## Results

In addition to providing evidence for the streamlines that originated from bilateral Crus ROIs and projected in Broca’s complex, we found reciprocal structural network interactions between these two areas with unexpected characteristics *via* all tractography results obtained *via* these three separate methods.

### Delineation of Characteristics of Tracts Through Subject- and Group-Level Visual Inspections

**FIGURE 3:**
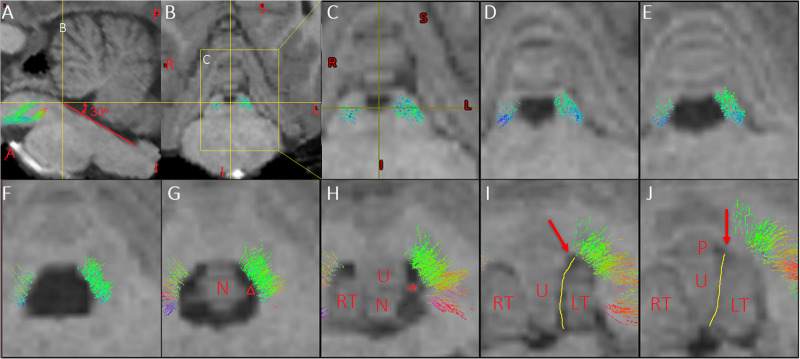
Multi-slice views of the pathway of Crus–Broca tracts related to the fourth ventricle and supratonsillar cleft. **(A)** In a sagittal slice, the focus point locates on the inferior border of the aqueduct. The angles between the yellow horizontal line and the red line superimposed over the floor of the fourth ventricle is about 30°, which is the mean operative angle of the telovelar approach (TeA) targeting the aqueduct reported in Figure 9B of one anatomical study (Tanriover et al., 2004). Line B demonstrates the coronal level of the coronal slice in **(B)**. **(B)** The yellow box demonstrates the location of a slice in **(C)**. **(C–H)** In the anatomical directionality from rostral to caudal, multi-slice views demonstrate the spatial relationship between the pathway of the Crus–Broca tracts and the fourth ventricle and supratonsillar cleft in the simulating angle of view of the TeA targeting the aqueduct. **(I,J)** Red arrows indicate the uvula–pyramidal junction which is the broadest strip of vermis and limits the lateral retraction of the tonsils and the operative angle of the TeA. The yellow lines indicate the uvulotonsillar cleft ending superiorly at the uvula–pyramidal junction, which is often opened to resect large tumors in the rostral part of the fourth ventricle (Rajesh et al., 2007). **(G,H)** After the opening the uvulotonsillar cleft, the lateral retraction of the tonsils and the medial retraction of the uvula and the nodule, one entryway to the rostral, dorsal, and lateral aspects of the fourth ventricle is obtained through the supratonsillar cleft (indicated by “△”). Of note is that, in these two slices, the pathway is next to the lateral bank of the entryway. The asterisk indicates the superior pole of the left tonsil **(C–G)**. These streamlines next to the superolateral roof of the fourth ventricle should be protected from damage during tumor resection. LT, left tonsil; N, nodule; P, pyramid; RT, right tonsil; U, uvula.

**FIGURE 4:**
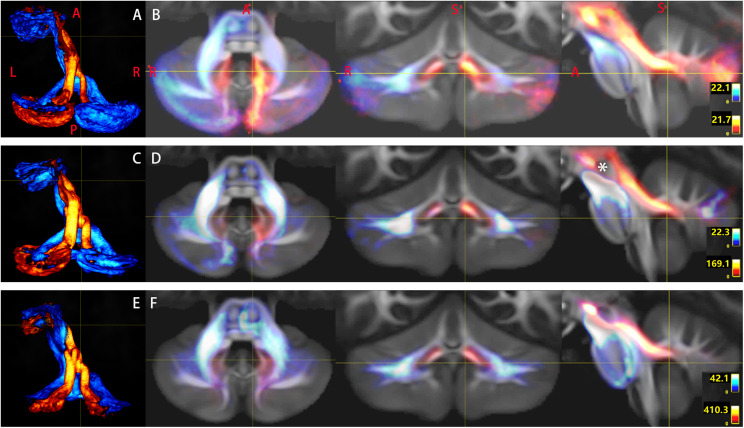
Tridimensional views of the pathways of Crus–Broca tracts (hot colors)/Broca–Crus tracts (cold colors) from all subjects as reconstructed by DSI Studio **(A,B)**, FSL **(C,D)**, and MRtrix3 **(E,F)**. **(A,C,E)** Pathways of the two kinds of tracts. **(B,D,F)** (1) Crus–Broca tracts are closer to the uvula and the pyramid than the dentate nucleus (DN) is and are closely above the superior border of the supratonsillar cleft; (2) Crus–Broca tracts bypass the left red nucleus; and (3) distributions of origin points of Crus–Broca tracts and target points of Broca–Crus tracts in the medial and the exterior part of Crus regions of interest, respectively. Each image shows Crus–Broca tracts/Broca–Crus tracts from all subjects by interactively resetting the image scaling to optimally display part of the Crus–Broca tracts that go through the DN and superior cerebellar peduncle. The asterisk in **(D)** indicates the left red nucleus. The focus point in **(B,D,F)** indicates the supratonsillar cleft.

**FIGURE 5:**
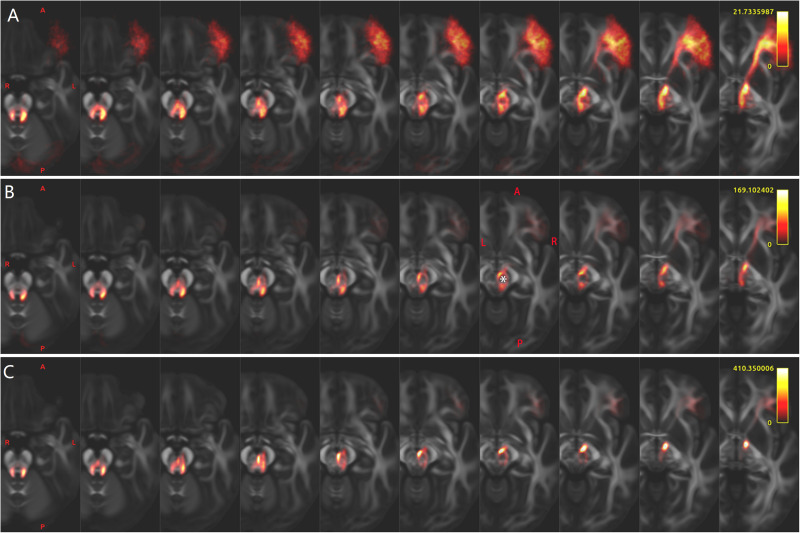
Multi-slice views of the pathway of Crus–Broca tracts that bypass the left red nucleus (RN). The pathways of Crus–Broca tracts from all subjects reconstructed *via* DSI Studio **(A)**, FSL **(B)**, and MRtrix3 **(C)** are consistent with one another. The decussating paths originating from the right Crus regions of interest (ROI)s merge with the non-decussating paths from the left Crus ROIs into one fasciculus, which bypasses the left RN (denoted by the white asterisk in the seventh image of **B**). The anatomical directionality of the multi-slice images from left to right for each panel is from inferior to superior.

**FIGURE 6:**
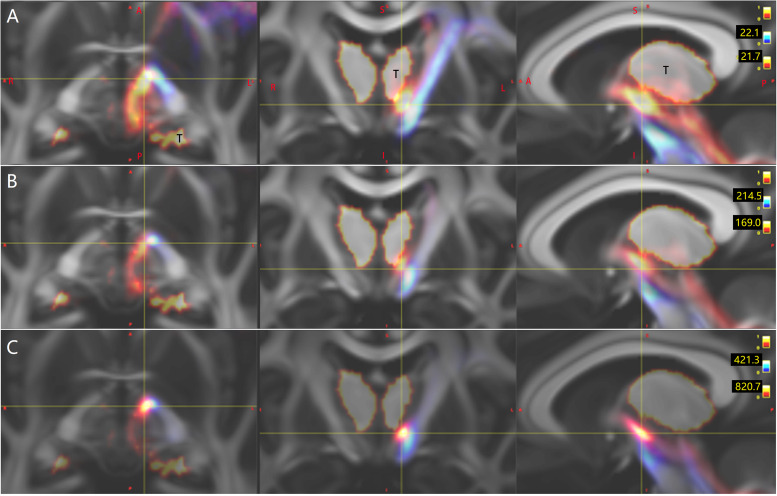
Tridimensional views of the pathways of Crus–Broca tracts that bypass the left thalamus. The pathways of Crus–Broca tracts (hot color)/Broca–Crus tracts (cold color) from all subjects as reconstructed *via* DSI Studio **(A)**, FSL **(B)**, or MRtrix3 **(C)**. At the group level, Crus–Broca tracts bypass the left thalamus [denoted by “T” in **(A)**]. The focus in each image denotes the voxel cluster where the tracts most commonly go through but is outside of the left thalamus.

**FIGURE 7:**
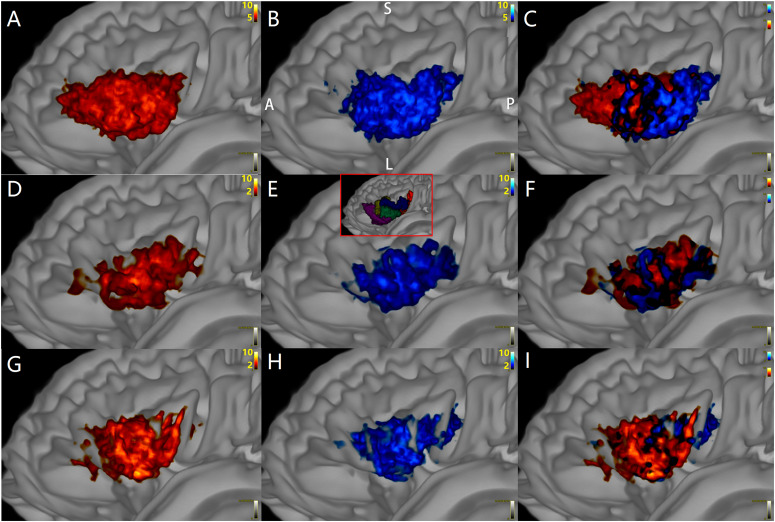
The distributions of target points (red) of Crus–Broca tracts and origin points (blue) of Broca–Crus tracts in Broca’s complex from all subjects. The central area of Broca’s complex is where the majority of target points (red) of Crus–Broca tracts and origin points (blue) of Broca–Crus tracts are located. The posterior part is more involved in Broca–Crus tracts. However, the anterior part is more involved in Crus–Broca tracts. The above-mentioned trend is across all tractography results *via* DSI Studio **(A–C)**, FSL **(D–F)**, and MRtrix3 **(G–I)**. The inset in **(E)** demonstrates the locations of five Broca ROIs.

Bilateral Crus–Broca tracts originated predominantly from the medial part of bilateral Crus ROIs with a leftward predominance, synapsed on the DN, exited the hilus of the DN, and ascended into the ipsilateral SCP (Figures 2–4). Then, their decussating tracts crossed at the same level of the mesencephalon across tractography methods, merged with their non-decussating tracts into one fasciculus, and surprisingly bypassed the left RN and the left thalamus (Figures 2–6). Specifically, just after exiting the hilus of the DN, compared to those of the DN, the Crus–Broca tracts were closer to the uvula and the pyramid and located closely superior to the superior border of the supratonsillar cleft and the superolateral roof of the fourth ventricle (Figures 3, 4). In Figure 3, the spatial relationship between the pathway of Crus–Broca tracts and the fourth ventricle and the supratonsillar cleft is shown from angle of view to target the aqueduct in the mean operative angles of 30°(through TeA as reported in Figure 9B of Tanriover et al. (2004). Through merging all subjects’ Crus–Broca tracts or Broca–Crus tracts within the same category of tract in a common space, we found that the central area of Broca’s complex was where the majority of target points (red) of Crus–Broca tracts and origin points (blue) of Broca–Crus tracts were located and that the posterior part was more involved in Broca–Crus tracts. However, the anterior part was more involved in Crus–Broca tracts (Figure 7). The Broca–Crus tracts descended into the ipsilateral or contralateral MCP and finally terminated predominantly at the exterior part of the bilateral Crus ROIs with right-side predominance (Figure 4).

Panel A shows that the distribution patterns of the target points (hot color) of the left-Crus–Broca tracts (i.e., 10 tracts from the left Crus ROIs to Broca ROIs; for example, 11–74 tract) in five Broca ROIs were not obviously different from those (jet color) of right-Crus–Broca tracts (i.e., 10 tracts from the right Crus ROIs to Broca ROIs; for example, 13–74 tract).

**FIGURE 8:**
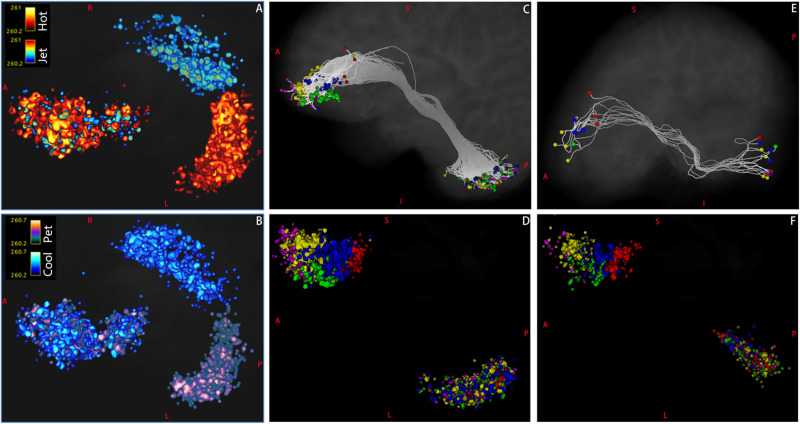
The distributions of endpoints of Crus–Broca tracts and Broca–Crus tracts as reconstructed *via* MRtrix3. **(A)** The distribution patterns of target points (in matlab_hot colormap) of the left Crus–Broca tracts in five Broca regions of interest (ROIs) are not obviously different from those (in matlab_jet colormap) of the right Crus–Broca tracts. **(B)** This trend is true in the origin points of Broca–left-Crus tracts (in matlab_pet colormap) and Broca–right Crus tracts (in matlab_cool colormap) in five Broca ROIs. **(C)** In one representative subject (LS 2043), after the endpoints of left Crus II–Broca tracts (five tracts) have been colored by the colors of their target Broca ROIs in Figure 1B, the origin points of these five tracts mix with each other in the left Crus II ROI rather than locate at exclusive subregions of the left Crus II ROI according to their target Broca ROIs. This trend is true in left Crus II–Broca tracts from all subjects **(D)**, Broca–right Crus II tracts from the same subject **(E)**, and Broca–right Crus II tracts from all subjects **(F)**. The color bar in each panel of the figure is from 0.2 to 0.5. The right two voxel clusters in **(A,B)** locate within bilateral Crus II ROIs, and the right voxel cluster in **(C,D)** locates within the left Crus II ROI; the right voxel cluster in **(E,F)** locates within the right Crus II ROI. The left voxel cluster in each panel locates within Broca ROIs.

**FIGURE 9:**
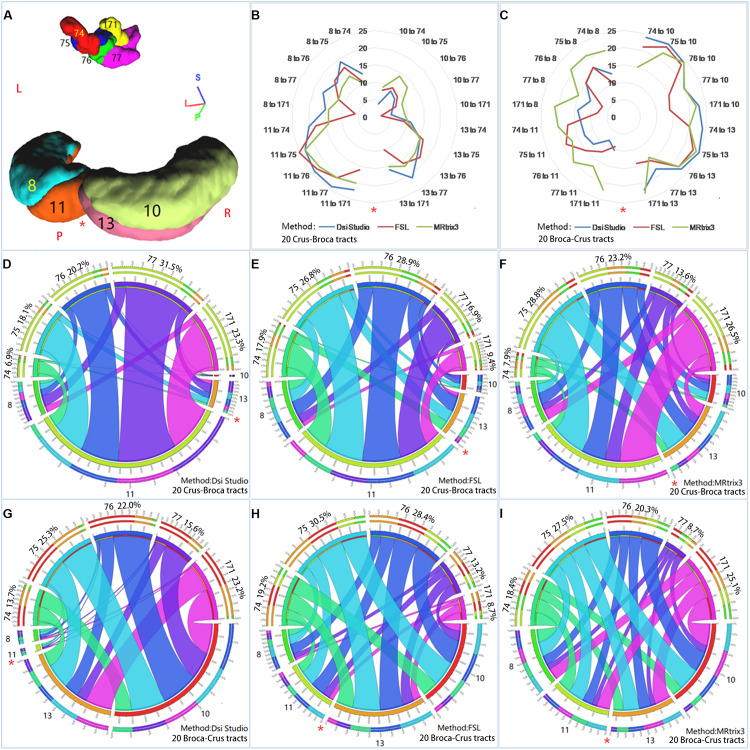
Reciprocal structural network interactions between bilateral Crus lobes and Broca’s complex. **(A)** Bilateral Crus lobes [four Crus regions of interest (ROIs)] and five ROIs of Broca’s complex (five Broca ROIs) used to reconstruct reciprocal structural network interactions. **(B,C)** The numbers of subjects among the 26 subjects for which each of 20 Crus–Broca tracts **(B)** or each of 20 Broca–Crus tracts **(C)** are reconstructed *via* each tractography method. As the tool of “Circos” show, reciprocal structural network interactions between these Crus ROIs and Broca ROIs through Crus–Broca tracts **(D–F)** and Broca–Crus tracts **(G–I)** exist by adding the structural connectivity strength values of each Crus–Broca tract and Broca–Crus tract from all subjects together within the same category of tract as reconstructed by one method among DSI Studio **(D,G)**, FSL **(E,H)**, and MRtrix3 **(F,I)**. The asterisk in each panel indicates the midline separating the left Crus ROIs from the right Crus ROIs. Each number following the name of Broca ROI (for example, “74”) in each panel is the percentage of the ROI’s contribution as target points in Crus–Broca tracts **(D–F)** and also as origin points in Broca–Crus tracts **(G–I)**.

Through the “tckmap” tool of MRtrix3, we found that, at the group level, the distribution patterns of the target points of left-Crus–Broca tracts (i.e., 10 tracts from the left Crus ROIs to Broca ROIs; for example, 11–74 tract) in the five Broca ROIs were not significantly different from those of right-Crus–Broca tracts (i.e., 10 tracts from the right Crus ROIs to Broca ROIs; for example, 13–74 tract; Figure 8A). These results were not rejected by visual inspection of Figures 9D–F in which the proportions between the numbers of “streamlines” that each Broca ROI received from the left Crus ROIs and from the right Crus ROIs were not obviously different. This trend was true in the origin points of Broca–left-Crus tracts and Broca–right Crus tracts (Figures 8B, 9G–I). More interestingly, the origin points of Crus–Broca tracts originating from one Crus ROI mixed with each other in the Crus ROI rather than being located at exclusive subregions of the Crus ROI according to their target Broca ROIs. The target points of Broca–Crus tracts to one Crus ROI also mixed with each other in the Crus ROI rather than being located at exclusive subregions of the Crus ROI according to their origin Broca ROIs (Figure 8E, taking the tracts connecting five Broca ROIs and the left Crus II ROI for instance; see Figures 8C–F).

### Delineation of Characteristics of Tracts *via* Direct Analysis of Their SCSs

According to our criterion for whether one tract was reconstructed, the numbers of data sets from subjects in which each left-Crus–Broca tract was reconstructed by each tractography method were more than those of each right-Crus–Broca tract. For example, the 11–75 tract was reconstructed in 25 data sets of subjects *via* FSL (red line), whereas the 10–171 tract was only reconstructed in two data sets of subjects *via* FSL (Figure 9B). However, the trend was the opposite for Broca–Crus tracts. For example, the 75–10 tract was reconstructed in 25 data sets of subjects *via* DSI Studio (blue line), whereas the 77–8 tract was only reconstructed in seven data sets of subjects *via* FSL (Figure 2C). The sum of the SCS values of the left-Crus–Broca tracts was greater than that of the right-Crus–Broca tracts in data from 24, 24, and 20 subjects reconstructed *via* DSI Studio, FSL, and MRtrix3, respectively. Correspondingly, data from 23, 24, and 15 subjects’ sums of SCS values of Broca–right Crus tracts (10 tracts) were greater than those of Broca–left-Crus tracts (10 tracts).

All subjects’ SCS values of each Crus–Broca or Broca–Crus tract were added together within the same category of tract to show the reciprocal structural network interactions between Crus ROIs and Broca ROIs (Figure 9A) *via* the tool of “Circos”^4^. We found that each Crus ROI sent/received streamlines to/from each Broca ROI and each Broca ROI sent/received streamlines to/from each Crus ROI with varying strengths (Figures 9D–I).

HCP 75 almost always had the biggest percentage (see the numbers following the names of Broca ROIs in each panel of Figure 9) of the contribution as target points in Crus–Broca tracts (Figures 9E,F) and also as origin points in Broca–Crus tracts (Figures 9G–I), except in Crus–Broca tracts from DSI Studio (Figure 9D). In the tracts from DSI Studio, the percentages of the contributions of HCP 74, HCP 75, and HCP 76 as origin points in Broca–Crus tracts were more than those of the target points in Crus–Broca tracts, whereas the trend was the opposite for HCP 77 and HCP 171 (Figures 9D,G). This pattern was also seen in the tracts from FSL (HCP 74 and HCP 75 vs. other Broca ROIs; Figures 9E,H) and also MRtrix3 (HCP 744 vs. other Broca ROIs; Figures 9F,I). In terms of Crus ROIs, each left Crus ROI sent more streamlines to each Broca ROI than each right Crus ROI did and received less streamlines from each Broca ROI than each right Crus ROI did. Therefore, a left asymmetry of Crus–Broca tracts and a right asymmetry of Broca–Crus tracts were demonstrated not only in the left vs. the right Crus ROIs but also in each Broca ROI, although the extent of these two asymmetries varied from high to low in the order of DSI Studio, FSL, and MRtrix3 (Figures 9D–I).

### Delineation of Characteristics of Tracts *via* Statistical Analysis

**FIGURE 10:**
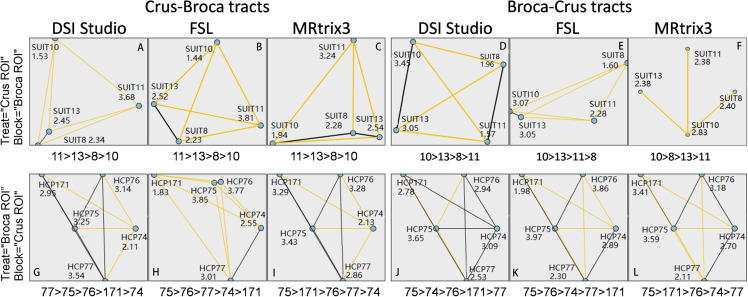
Statistical analysis of contributions of each Broca region of interest (ROI) (or each Crus ROI) in Broca–Crus tracts or Crus–Broca tracts by comparing the mean ranks of the structural connectivity strength (SCS) values of these tracts grouped by Broca ROIs (or Crus ROIs). In terms of Crus ROIs (i.e., treat = “Crus ROI”), Crus–Broca tracts from each tractography method originate more from the left Crus ROIs than from the right Crus ROIs (SUIT 11 > SUIT 13 > SUIT 8 > SUIT 10) **(A–C)**, whereas Broca–Crus tracts from DSI Studio/FSL and Mrtrix3 project more to the right Crus ROIs than to the left Crus ROIs [SUIT 10 > SUIT 13 > SUIT 11 > SUIT 8 **(D,E)** and SUIT 10 > SUIT 8 > SUIT 13 > SUIT 11 **(F)**]. In terms of Broca ROIs (i.e., treat = “Broca ROI”), the results are not as consistent as those of Crus ROIs. However, HCP 75 (BA 45) ranks first among those receiving “streamlines” of Crus–Broca tracts **(G–I)** or among those sending “streamlines” of Broca–Crus tracts **(J–L)** [the black line connecting the points of HCP 77 and HCP 75 in **(G)** denotes that no significant difference exists between their rankings]. In **(A)**, Crus–Broca tracts reconstructed *via* DSI Studio were divided into four groups based on their origin Crus (SUIT) ROIs. These numbers represent the mean ranks of the SCS values of each group with their target Broca ROIs as “block” factors.

No significant differences were found between female and male subjects’ SCS values of each Broca–Crus/Crus–Broca tract obtained by any method. The CC (95% CI) of different methods when defining the SCS values of Crus–Broca tracts (n = 520) was 0.43 (0.36 0.50) with MRtrix3 and DSI Studio, 0.34 (0.26 0.41) with MRtrix3 and FSL, and 0.52 (0.45 0.59) with DSI Studio and FSL. Additionally, in terms of Broca–Crus tracts, the CCs (95% CIs) were 0.32 (0.24 0.39) with MRtrix3 and DSI Studio, 0.29 (0.21 0.37) with MRtrix3 and FSL, and 0.42 (0.35 0.49) with DSI Studio and FSL when defining the SCS values of BrocaŰCrus tracts (n = 520). All the original *p* values (two-tailed) of Spearman rank–order correlation tests were shown as 0.000, which survived from the FDR procedure and indicated the statistical significance of these CCs. As shown in Figure 10, in terms of Crus ROIs, the Crus–Broca tracts from each tractography method originated more from the left Crus ROIs than from the right Crus ROIs (SUIT 11 > SUIT 13 > SUIT 8 > SUIT 10), whereas the Broca–Crus tracts from DSI Studio/FSL and Mrtrix3 projected more to the right Crus ROIs than to the left Crus ROIs (SUIT 10 > SUIT 13 > SUIT 11 > SUIT 8 and SUIT 10 > SUIT 8 > SUIT 13 > SUIT 11), indicating the left asymmetry of Crus–Broca tracts and the right asymmetry of Broca–Crus tracts. In terms of Broca ROIs, the results were not as consistent as those of Crus ROIs. However, the two following points indicated the consistency among related tractography results derived from each method. The analysis demonstrated that HCP 75 (BA 45) sent/received maximum “streamlines” of Broca–Crus/Crus–Broca tracts (in the case of Broca–Crus tracts reconstructed *via* DSI Studio, although HCP 77 ranked before HCP 75, no significant difference existed in their rankings). Based on the above-mentioned results, Kendall’s coefficient of concordance (k sample) analysis demonstrated that the degree of agreement of all three tractography methods in ranking the differential contributions of Broca ROIs and Crus ROIs in Broca–Crus tracts or Crus–Broca tracts could not be rejected. The left asymmetry of Crus–Broca tracts or the right asymmetry of Broca–Crus tracts reconstructed *via* each method was supported *via* related-samples Wilcoxon signed-rank tests by comparing the SCS values of Crus–Broca tracts originating from the left Crus ROIs with those from the right Crus ROIs or by comparing the SCS values of Broca–Crus tracts projecting in the right Crus ROIs with those in the left Crus ROIs (the original *p*-values of all tests were shown as 0.000, which survived the FDR procedure.). Their 95% CIs of the median of SCS values of all the Crus–Broca tracts, as well as the Broca–Crus tracts, defined by bootstrapping indicated that both asymmetries were in the 8- to 9-year-old group than those in all subjects (Table 1).

**TABLE 1:** Statistical analysis of the structural connectivity strength (SCS) values of the analyzed tracts indicates a left asymmetry of Crus–Broca tracts and a right asymmetry of Broca–Crus tracts.

	SCS values of Crus–Broca tracts originating from the left Crus regions of interest (ROIs) (sample 1) vs. that from the right Crus ROIs (sample 2)			SCS values of Broca–Crus tracts projecting in the right Crus ROIs (sample 1) vs. that in the left Crus ROIs (sample 2)		
Fiber tracking method *via* which the tracts were reconstructed	DSI Studio	FSL	Mrtrix3	DSI Studio	FSL	Mrtrix3
95% confidence interval (CI) of the median of the difference between these two samples of all subjects*	13.0 (9.0, 18.0)	332.0 (243.0, 408.0)	1.5 (1.0, 2.0)	20.0 (27.0, 34.0)	303.5 (237.0, 374.0)	0.5 (0.0, 1.0)
95% CI of the median of the difference between these two samples of group 8 to 9 years*	15.5 (8.5, 17.5)	361.0 (220.0, 502.0)	9.5 (6.5, 12.5)	91.8 (49.0, 207.0)	541.8 (366.0, 704.5)	1.5 (1.0, 3.0)

*95% CI of the median of the difference between each of two samples was determined by the Hodges–Lehmann 95% CI test.

## Discussion

### Reciprocal Structural Connections Between Bilateral Crus Lobes and Broca’s Complex

The clinical imaging of patients with cerebellar disorders has revealed a broad range of motor speech impairments in terms of both execution and planning (Marien et al., 2006; De Smet et al., 2007; Marien and Verhoeven, 2007; Korah et al., 2010; Di Rocco et al., 2011; De Smet et al., 2013; De Witte et al., 2017). Several studies have found that cerebellar internal models, mainly in the right posterolateral cerebellum, transmit predictions on coming ‘non-motor’ information in several linguistic processes of phonology, semantics, syntax, and others (Miall et al., 2016; Runnqvist et al., 2016; D’Mello et al., 2017; Lesage et al., 2017). Associative memory traces of contiguous linguistic events, including the processing of the phonological, semantic, and syntactic properties of words, are stored in the cerebellum and will be used later (Argyropoulos, 2016). *Via* a dense network of cerebro-ponto-cerebello-cerebral pathways, the linguistic internal models stored in the cerebellum receive a copy in particular from the fronto-temporal input analysis system and convey to the prefrontal cortex (Marien and Manto, 2018). Hence, understanding related anatomical connection is important to elucidate the cerebellum’s contribution to speech processing. Two studies reported evidence for the decussate streamlines originating from Broca’s area and projecting into the cerebellum as part of their reconstructed pathway (Palesi et al., 2017; Pieterman et al., 2017). Recently, Ji et al. (2019) used seed ROI in the DN and target ROI in the contralateral frontal cortex to reconstruct the decussate cerebello-thalamocortical pathway, which included those projecting into BA 44 and BA 45. In the present study focused on structural connections between the left IFG and the cerebellum, the tractography results showed reciprocal structural network interactions between bilateral Crus lobes and Broca’s complex which, in the above-mentioned context, were not unreasonable but needed to be verified.

In addition to cerebellar internal models related to language mainly stored in the right posterolateral cerebellum as mentioned above, many recent studies demonstrated a division of labor for phonological, syntactic, and semantic planning in Broca’s complex (Flinker et al., 2015; Glasser et al., 2016; Long et al., 2016; Belyk et al., 2017; Giahi Saravani et al., 2019; Klaus and Hartwigsen, 2019). If verified, the existence of reciprocal structural connections between bilateral Crus lobes and Broca’s complex would (1) give credible evidence to support a direct role of the bilateral cerebellums in motor speech planning and (2) explain why AoS can occur in patients with etiologically heterogeneous cerebellar disorders (Marien et al., 2006; De Smet et al., 2007; Marien and Verhoeven, 2007) and after pediatric MB surgery (De Witte et al., 2017). In the latter case, damage of efferent cerebellar pathways, such as part of the Crus–Broca tracts, would give rise to AoS due to defect of output of cerebellar internal models predicting and consequently optimizing phonological planning and execution (Marien and Manto, 2018).

It was thought that the cerebellum may influence the neocortex in a reciprocal fashion of one cerebellar region connecting with only one cortical region (Kelly and Strick, 2003). Interestingly, the tractography results showed reciprocal structural network interactions between bilateral Crus lobes and subregions of Broca’s complex and the origin points/target points of Crus–Broca tracts/Broca–Crus tracts in each Crus lobe mixing with each other rather than being located at exclusive subregions of the Crus lobe according to their target/origin Broca ROIs. How to interpret these results in respect of computation patterns in Crus lobes’ participating in the language function of Broca’s complex needs more evidence.

### Crus–Broca Tracts Bypass the Left RN and the Left Thalamus

It was reported that the cerebellum projects to the dorsolateral prefrontal cortex (not including IFG) and the primary motor cortex through the ventrolateral thalamic nucleus and other thalamic nuclei, including the mediodorsal thalamic nucleus in non-human primates (Middleton and Strick, 2001; Kelly and Strick, 2003). Therefore, our ROI approach for the Crus–Broca tracts only limited the tractography processes with cerebral ROIs, cerebellar ROIs, and the SCP. The tractography results showed that the pathway of these tracts was consistent across different methods in which these tracts traversed through the DN but bypassed the left RN and the left thalamus. In terms of the pathway of the Broca–Crus tracts, although our ROI approach for this kind of tract did not include pontine ROIs, their pathways as shown in the present study did not differ from those described in previous studies (Palesi et al., 2017; Pieterman et al., 2017), which suggested that our ROI approaches had the capacity to untangle complex conditions (such as long-distance and crossing fibers) in the cerebro-cerebellar loop.

It has been accepted that, for ultimately influencing the neocortex, the outputs of Purkinje cells first directly influence the thalamus *via* projections from deep cerebellar nuclei (Diedrichsen et al., 2019). Hence, recent studies have often used the RN (Palesi et al., 2015; Jakab et al., 2016; Fenoy et al., 2017; Schlaier et al., 2017; Petersen et al., 2018; Jang and Kwon, 2019) and/or the thalamus (Kwon et al., 2011; Surova et al., 2015; Bernard et al., 2016; Jakab et al., 2016; Meola et al., 2016; Mollink et al., 2016; Fenoy et al., 2017; Oh et al., 2017; Pieterman et al., 2017; Petersen et al., 2018; Jang and Kwon, 2019) as a ‘waypoint region’ to reconstruct the streamlines originating from the cerebellum and projecting in the cerebrum *via* tractography, but none of them reported the streamlines originating from the cerebellum and projecting in the left IFG. As mentioned previously, Ji et al. (2019) reconstructed the decussate cerebello-thalamocortical pathway, including those projecting into BA 44 and BA 45, without using the RN or the thalamus as a “waypoint mask.” They declare that 85 to 98% and 100% of the whole reconstructed pathway passed through the contralateral RN and contralateral thalamus, respectively. Unfortunately, the authors never refer to the method used for obtaining this result and no image material was used to display the exact spatial relationships between the whole pathway and the RN or the thalamus, let along between the cerebellar streamlines projecting into the BA 44 and BA 45 and the RN or the thalamus (Ji et al., 2019). If our findings about the trajectory of the Crus–Broca tracts were true, then the ROI approach with the thalamus or the RN as a “waypoint mask” used for reconstruction of all cerebello-cerebellar tracts for tract-based analysis would result in at least the Crus–Broca tracts excluded.

### The Potential Impact of These Findings on the Prevention of CMS

Disruption of cerebellar outflow tracts has been suggested to play a key role in the pathogenesis of CMS (Morris et al., 2009; Renne et al., 2019). These tracts can be secondly damaged during improper splitting of the cerebellar vermis through TrA or improper manipulating of the superior border of the supratonsillar cleft due to large and midline MB through TeA (Tanriover et al., 2004; Rajesh et al., 2007; Akakin et al., 2014). In our preliminary experiments that reconstructed streamlines originating from the cerebellum and projecting in the left IFG using MRtrix3 software, we found that the vast majority of these cerebello-cerebral streamlines originated from four bilateral Crus lobes rather than from the inferior vermis (these data will be published later). After exiting the hilus of the DN, the Crus–Broca tracts were closer to the uvula and the pyramid than the DN was and were closely superior to the superior border of the supratonsillar cleft; this border limits the operative angle to the superior part of the fourth-ventricle roof (Tanriover et al., 2004) and next to the superolateral roof of the fourth ventricle [Figures 3, 4 in the present study, Figure 7H in Akakin et al. (2014), and Figure 7A in Jittapiromsak et al. (2010))]. In terms of the Crus–Broca tracts that may represent one of the important edges of motor speech networks, it may be more important to spare the bilateral DN and the SCP compared to the inferior vermis itself during surgery for the prevention of CMS. Of note is that the trajectory of the Crus–Broca tracts around the fourth ventricle that we visualized in fact represented that of the bilateral SCP. During resection of large tumors *via* TeA, after the opening of the uvulotonsillar cleft, the lateral retraction of the tonsils, and the medial retraction of the uvula and nodule, one entryway to the rostral, dorsal, and lateral aspects of the fourth ventricle is obtained through the supratonsillar cleft, keeping in mind that these crucial tracts are next to the lateral bank of the entryway and the superolateral roof of the fourth ventricle (Figure 3).

### Left Asymmetry of Crus–Broca Tracts and Right Asymmetry of Broca–Crus Tracts

One recent study, including 232 (74.8%) subjects with left-hemispheric lateralization and 78 (25.32%) with right-hemispheric or bilateral lateralization, refuted a single monogenetic model for degree of hand preference and degree of language lateralization (Somers et al., 2015). There were only three left-handed subjects (scoring 0, 1, and 3) included in the present study. Furthermore, among the eight subjects with minimum sums of SCS values of the Crus–Broca tracts as determined by MRtrix3, seven were 100% right-handed. Therefore, a statistical analysis for it was not performed. Our findings supported the principle from animal studies (Strick et al., 2009) that the major source of cerebral cortex (here it is BA 45:HCP 75) as inputs to cerebro-cerebellar fibers is the major target of the cerebral cortex (here it is also BA 45) as outputs from the cerebello-cerebral fibers. However, in terms of the endpoints of the Crus–Broca loops in the cerebellums, the largest source of cerebellar input to the Crus–Broca tracts was the left Crus II and the largest target of cerebellar output from the Broca–Crus tracts was the right Crus I, which meant the left asymmetry of the Crus–Broca tracts and the right asymmetry of the Broca–Crus tracts. To our knowledge, it was the first time to propose the existence of the left-Crus–Broca tracts and the left- Broca–Crus tracts here, both of which were non-decussate cerebello-cerebral tracts.

There are literatures showing clear evidence of the right laterality of cerebellums in language function (Marien and Borgatti, 2018; Stoodley and Schmahmann, 2018) and suggesting the right efferent cerebellar pathway to be likely more significantly involved in the pathophysiology of CMS (Law et al., 2012; Avula et al., 2016). Recently, we have found that, to reconstruct the decussate dentatothalamocortical tracts and the non-decussate counterparts (the latter have been reported in recent literatures; Keser et al., 2015; Karavasilis et al., 2019) with MRtrix3 using the same seed number, respectively, the former reconstructed more streamlines than the latter did (these results will be published later). In this study, the left (non-decussate) Crus–Broca tracts and the right (decussate) Crus–Broca tracts were tracked with the same strategy. Considering that the decussate Crus–Broca tracts go through the decussation of SCP but the non-decussate ones do not and there was no other evidence to support the left asymmetry of the Crus–Broca tracts, it would be better to interpret this result from the view of different fiber-tracking difficulty of the decussate and the non-decussate Crus–Broca tracts. By contrast, the decussate Broca–Crus tracts do not have this kind of crossing-fiber concentrated area to go through, and results from each tractography method showed the right asymmetry of the Broca–Crus tracts.

While clear evidence shows the right laterality of the cerebellums in language function, tasks involving language-related processing are defined in the bilateral Crus lobes (King et al., 2019, Nat Neurosci). Cohen et al. (2009) reported one patient suffering from foreign accent syndrome (FAS), a subtype of AoS, after a left parietal stroke. Interestingly, a second right cerebellar stroke that happened to the patient paradoxically resolved FAS rather than further impairing speech. The authors speculated that competitive interactions between both cerebellar hemispheres was involved in controlling the rhythmic and the prosodic patterns of speech and argued that, after right cerebellar damage, the disinhibited left cerebellar hemisphere took over the functional role in motor speech production (Cohen et al., 2009). Our findings about the bilateral Crus lobes sending streamlines to the left extended Broca’s complex would provide new evidence to support the notion of the bilateral cerebellums participating in motor speech planning. More importantly, the present study provided one option of ROI approach and acquisition protocol of dRMI, both of which had been used to successfully reconstruct bilateral Crus–Broca tracts; for track-based analysis of a future clinical image to explore, for example, whether injury to the right efferent cerebellar pathway more likely causes CMS, the greater SCS value of the left- Crus–Broca tracts suggests better tolerance to injury of the right ones.

### Limitations of the Present Study and Future Work

The reliability of the tractography results is inferior to that of anatomical dissections or tract tracing. However, in terms of language-related cerebro-cerebellar loop, tract tracing may be unable to target analogous structures in animal models and anatomical dissections may not be able to resolve their long and crossed pathways in humans. In this case, we used tractography, the only existing method for tracking axonal fibers in humans *in vivo* to explore the structural connection between these two regions; however, our results highly need verification. Concerning the age of common patients with CMS, we preferentially selected HCP-Lifespan data, which includes high-quality data from children not easily available for us at that time. However, it only included 26 subjects of varying age groups, which lowered the credibility of the results. Therefore, we will publish the results of comparing the outcomes of the present study with those derived from higher-quality dMRI data of other HCP projects in the recent future. Even if the existence of the Crus–Broca loops was verified, it would be among the most important edges of motor speech networks. Therefore, interpretation of these results in the context of structural connections between all cerebral cortices and bilateral cerebellar cortices is being demanded. In terms of whether or not our results may ultimately contribute to the treatment and/or prevention of CMS, clinical studies with a relatively larger sample size are required.

## Conclusion

Through tractography, we provided evidence of the reciprocal structural network interactions between the bilateral Crus lobes and five ROIs of Broca’s complex with varying strengths. If verified, these findings would be suggestive of the bilateral cerebellums’ role in motor speech planning and new clue to prevent CMS.

## Data Availability Statement

The datasets analyzed in this study are publicly available structural MRI (sMRI) and dMRI imaging data from the HCP Lifespan Pilot project – 1a (https://db.humanconnectome.org/data/projects/WU_L1A_Cur).

## Ethics Statement

Ethical review and approval was not required for the study on human participants in accordance with the local legislation and institutional requirements. Written informed consent to participate in this study was provided by the participants’ legal guardian/next of kin.

## Author Contributions

YW and HZ conceived and designed the experiments. HZ, YB, YF, and HH performed the experiments. HZ and YF conducted the statistical analysis. HZ and YW contributed in the data interpretation and figure preparation. HZ, YB, YF, and YW wrote and revised the manuscript.

## Conflict of Interest

The authors declare that the research was conducted in the absence of any commercial or financial relationships that could be construed as a potential conflict of interest.

## Footnotes

1 https://db.humanconnectome.org/data/projects/WU_L1A_Cur

2 https://humanconnectome.org/storage/app/media/documentation/lifespan-pilot/WU-Minn_HCP_LifespanPilot_Report_10Feb2015.pdf

3 http://dsi-studio.labsolver.org

4 http://mkweb.bcgsc.ca/tableviewer/visualize/

## Abbreviations

AoS: apraxia of speech
CC: correlation coefficient
CI: confidence interval
CMS: cerebellar mutism syndrome
DN: dentate nucleus
IFG: inferior frontal gyrus
MB: medulloblastoma
MCP: middle cerebellar peduncle
RN: red nucleus
ROI: region of interest
SCP: superior cerebellar peduncle
SCS: structural connectivity strength
SUIT: spatially unbiased atlas for the cerebellum and brainstem
TDI: track-density imaging
TeA: telovelar approach
TrA: transvermian approach.
